# Valorization of Traditional Alcoholic Beverages: The Study of the Sicilian Amarena Wine during Bottle Aging

**DOI:** 10.3390/foods11142152

**Published:** 2022-07-20

**Authors:** Giuseppa Di Bella, Miriam Porretti, Ambrogina Albergamo, Claudio Mucari, Alessia Tropea, Rossana Rando, Vincenzo Nava, Vincenzo Lo Turco, Angela Giorgia Potortì

**Affiliations:** 1 Department of Biomedical, Dental, Morphological and Functional Images Sciences (BIOMORF), University of Messina, 98100 Messina, Italy; gdibella@unime.it (G.D.B.); rrando@unime.it (R.R.); vnava@unime.it (V.N.); vloturco@unime.it (V.L.T.); agpotorti@unime.it (A.G.P.); 2 Department of Chemical, Biological, Pharmaceutical and Environmental Sciences, University of Messina, 98100 Messina, Italy; miriam.porretti@studenti.unime.it; 3 Fondazione Albatros—ITS Agroalimentare, 98100 Messina, Italy; claudio.mucari@gmail.com; 4 Department of Research and Internationalization, University of Messina, 98100 Messina, Italy; atropea@unime.it

**Keywords:** Amarena wine, sour cherry, fortified wine, traditional alcoholic beverages, oenological parameters, chromaticity, inorganic elements

## Abstract

Traditional alcoholic beverages have always been part of the Mediterranean culture and, lately, they have been re-evaluated to valorize both the territory and local customs. In this study, the Amarena wine, a fortified wine included in the national list of the traditional agri-food products, was characterized during bottle aging for oenological parameters, chromaticity, volatiles, and inorganic elements. Then, experimental data were visually interpreted by a principal component analysis (PCA). PCA revealed that most of oenological parameters (i.e., alcoholic grade, total dry extract, sugars, organic acids, and phenolic compounds) had a scarce discriminating power. Additionally, ethyl esters were only present in younger products, while remaining at quite constant levels. Conversely, certain metals (i.e., Mg, Na, Mn, Zn, and Cu), chromatic properties, and pH differentiated older Amarena bottles from the younger counterpart. Particularly, acetaldehyde and furanic compounds proved to be valid aging markers. A sensorial analysis highlighted that fruity and floral odors and flavors characterized younger beverages, while dried fruity, nutty, and spicy notes were displayed by older products, along with the valuable attribute of “oxidized” typically observed in aged Sherry wines. Overall, this study may encourage the production and commercialization of the Amarena wine, thus preserving the cultural heritage of the Mediterranean area.

## 1. Introduction

Due to peculiar nutritional, social, and religious implications, alcoholic beverages have certainly been a distinctive component of the Mediterranean culture [1] and, despite the spread of certain globalized consumption styles (i.e., so-called “binge-drinking”), the adherence to a healthier, light-to-moderate, alcohol consumption, is still evident throughout the Mediterranean area [2]. In Italy particularly, fermented drinks have typically underlined convivial and socializing meanings—being enjoyed during mealtimes in family and friend settings [3]—and have often shaped numerous regional gastronomies as well [4].

Even if Italy basically remains the country of wine drinkers—wine as a real food and not as an intoxicating drink [5,6]—there is a variety of traditional alcoholic beverages, such as distillates, liquors, aromatized and fortified wines, that are obtained by peculiar timeless production rituals based on the use of traditional local ingredients, such as parts or extracts from aromatic herbs or plants [1,7,8].

Although these preparations have been typically produced on a domestic scale and consumed at home or in the community, they have been lately revalued and placed on village and urban markets, and e-commerce as well [9]. Indeed, the consumption of traditional beverages would sensitize the modern consumer on current issues, such as the valorization of the territory and local customs [1,7], and the preservation of the traditional ecological knowledge and cultural diversity of the Mediterranean area [8].

Additionally, the principle “waste as a resource” already observed in many application areas [10,11,12,13,14,15,16,17,18,19,20,21,22,23,24,25,26], may also apply to such a sector, since the traditional beverage may be regarded as a circular and sustainable product obtained from the recovery and recycling of inexpensive and residual plant sources, thus avoiding the massive overharvesting of the species [8,27,28].

Most of the Italian traditional beverages own an added value, as they are in the list of Traditional Agri-food Products (TAPs) set by the Italian Ministry of Agriculture, Food and Forestry in collaboration with the Italian regions [29]. Among others, renowned TAP beverages are, for example, Annurca apple cider (Campania), bergamot liquor (Calabria), “Vino di Visciole” or “visner” (Marche), the “Elisir di China” (Toscana), nocino liquor and vin brulè (Emilia Romagna), and vermouth wine (Piemonte).

Sicily is among the regions with the highest number and variety of TAPs [30,31,32], including alcoholic products such as “Amarena”, a fortified wine traditionally prepared during the 1950s by rural communities from Valle del Nisi (province of Messina) on the autumnal grape harvest. The preparation owes its name to the employment of the leaves of *Prunus cerasus*, also known as sour cherry, a fruit plant probably native to Turkey that has subsequently spread in the Mediterranean area for the favorable climate conditions, becoming very common in central-southern Italy [33]. According to the original recipe from the nineteenth century, the beverage is prepared by cooking red grape must with sour cherry leaves and different aromas, including wood ash. Then, the preparation is fermented in oak barrel with fresh must and more sour cherry leaves for one year, decanted and, finally, aged in bottle for a period ranging from a few months to several years. Interestingly, before use, the sour cherry leaves should be macerated with fresh grape pomace until they reach a typical tobacco color.

A literature overview pointed out that the TAP beverages have been unfairly unexplored, as a marginal attention was just devoted to the chemical characterization of a limited number of products, such as the Annurca apple cider, citrus liquors and “Vino di Visciole” [34,35,36]. Hence, for the first time, the Sicilian Amarena wine was explored for oenological parameters, chromatic properties, inorganic elements, volatile profile, and sensorial properties. Given the renewed interest in protecting and valorizing the traditional alcoholic beverages, the characterization of the Sicilian Amarena wine would be of great interest to both producers and consumers to assess its overall quality, also in relation to bottle aging.

## 2. Materials and Methods

### 2.1. Samples

A total of 40 Amarena fortified wines produced by the family-owned company “Cantine De Francesco” from Valle di Nisi (Messina, Italy) were investigated. Over the investigated period, the traditional alcoholic beverage was always obtained by means of local unselected red grapes from the same vineyard according to the original recipe described above. After the first year in an oak barrel, the wine aging continued in 750 mL amber glass bottles closed with natural corks and kept into a cellar at the constant storage temperature of 14 ± 2.5 °C and humidity of 70 ± 3.5%.

For each aging period considered in the study, five bottles were selected and transported to the laboratory under refrigerated conditions. In the laboratory, all the bottles were opened for the first time and sampled, so that quintuplicate samples per bottle were obtained and stored at +4 °C until analysis. As a result, a total of 120 samples of fortified wines were considered. Further details of collected samples are reported in Table 1.

### 2.2. Material and Reagents

For the element analysis, suprapure grade nitric acid (HNO_3_, 65–69% *v*/*v*) and hydrogen peroxide (H_2_O_2_, 30% *v*/*v*), as well as ultrapure water (resistivity of 10 mΩ cm) were supplied by J.T. Baker (Mallinckrodt Baker, Milan, Italy). Commercial standard solutions of inorganic elements (i.e., Na, Mg, K, Fe, Cu, Mn, Zn, Cd, and Pb, 1000 mg/L in 2% HNO_3_, each) and volatiles (i.e., acetaldehyde, ethyl acetate, methanol, higher alcohols, furanic compounds and ethyl esters) were purchased from Supelco (Bellefonte, PA, USA). 

For the determinations of total polyphenols and anthocyanins, the Folin–Ciocalteu reagent was from Sigma-Aldrich (Steinheim, Germany). Methanol and water (HPLC grade) were provided by Carlo Erba (Val de Reuil, France), while ethanol (reagent grade, 96%, *v*/*v*), and commercial standard solutions of gallic acid and malvidin chloride were provided by Sigma-Aldrich.

### 2.3. Oenological Analysis

For every sample, alcoholic grade (% vol.), pH, total sugars (g/L), total acidity (g/L of tartaric acid), volatile acidity (g/L of acetic acid), and malic acid (g/L) were determined using the FOSS Wine-Scan equipped with WineScan software Version 2.2.1 (FOSS, Hillerod, Denmark), equipped with an FTIR (Fourier transform infrared spectroscopy) interferometer and an internal software (Foss Integrator) provided with built-in calibration curves.

Additionally, every sample was assessed for the content of total phenol (TPC) and total anthocyanins (TAC). TPC was assessed by a modified protocol based on the method proposed by Singleton and colleagues [37]. About 1 mL of the fortified wine sample was mixed with 5 mL of Folin–Ciocalteau reagent and 5 mL of a sodium carbonate solution (20%). The mixture was kept in the dark for 2 h and read at 760 nm with the UV-2401 PC spectrophotometer. A six-point calibration curve was constructed by means of different solutions with decreasing gallic acid concentrations. As a result, the total polyphenol content was calculated as mg of gallic acid equivalent per liter of beverage (mg GAE/L).

TAC was determined by the Ribereau-Gayon and Stonestreet method [38], based on the sodium bisulfite bleaching. In a glass tube, 250 μL of liquor sample were added with 250 μL of a 96% ethanol + 0.1% hydrochloric acid solution, 5 mL of 2% hydrochloric acid aqueous solution and 400 μL of distilled water. In a second tube, the same solution was added; however, the distilled water was replaced with 400 μL of 15% sodium bisulphite aqueous solution. After a 20 min incubation, the absorbance of every tube was measured at 520 nm using the UV-2401 PC spectrophotometer and the calculated difference corresponded to the free anthocyanin concentration, determined by reference to a six-point calibration curve of malvidin chloride. As a result, the TAC was reported as mg of malvidin equivalents per liter of fortified wine (mg ME/L).

For every type of analysis, triplicate analysis along with the relative analytical blanks were carried out.

### 2.4. Chromaticity Measurments

The chromatic characteristics of every fortified wine were determined by means of the protocol reported in the Commission Regulation (CEE) No. 2676/90 [39], setting the Community methods of analysis to be used in the wine sector. According to the Regulation, the chromatic traits of the alcoholic beverage are assessed by a spectrophotometric analysis in terms of luminosity (transmittance), which varies inversely with the color intensity, and chromaticity (dominant wavelength), which characterizes the hue and the purity of the color.

Briefly, every sample was clarified by centrifugation, and its optical path was measured with a UV-visible spectrophotometer (UV-2401 PC, Shimadzu, Milan, Italy) by recording the absorbances at 445 nm, 495 nm, 550 nm, and 625 nm. In parallel, the same optical path was defined for distilled water, as analytical blank to establish the zero of the absorbance scale. The absorbance values of the optical path were interpreted by a table of conversion factors to obtain the corresponding transmittances (T%), i.e., T_445_, T_495_, T_550_, and T_625_. Hence, the tristimulus *X*, *Y* and *Z* values were calculated as follows:

*X* = 0.42 T_445_ + 0.35 T_550_ + 0.21 T_625_

*Y* = 0.20 T_625_ + 0.63 T_550_ + 0.17 T_495_

*Z* = 0.24 T_495_ + 0.94 T_445._

On this basis, the luminosity was expressed by the *Y* value (%, for complete darkness: *Y* = 0%, for colorless liquids: *Y* = 100%). 

However, the *X, Y* and *Z* values were used also to calculate the chromaticity coordinates x and y as follows:

$$x=\frac{X}{X+Y+Z}$$

$$y=\frac{Y}{X+Y+Z}$$

Hence, the hue and purity of the sample were specified by the two derived parameters *x* and *y* plotted against a CIE *xy* chromaticity diagram. For every sample, triplicate measurements were performed.

### 2.5. Volatile Compounds

The extraction of volatiles occurred according to the method described by [40]. Briefly, the Amarena sample (~100 mL) was spiked with a known concentration of the internal standard (4-methylpentan-2-ol or octan-3-ol depending on the type of analyte) and stirred with 5 mL of dichloromethane for 1 h at room temperature. The organic layer was recovered through a separating funnel after 12 h at 2 °C, dried with anhydrous sodium sulphate, and stored at −20 °C until analysis.

The determination of acetaldehyde, ethyl acetate, methanol and higher alcohols was performed by a gas chromatography (GC) equipped with a split/splitless injector and a flame ionization detector (FID) system (Dani Master GC1000, Dani Instrument, Milan, Italy). The column was an Omegawax™ (30 m × 0.25 mm with a 0.25 µm film thickness, Supelco, Bellefonte, PA) and the temperature program was from 40 °C (3 min) to 180 °C (10 min) at 2 °C/min. The temperatures of injector and detector were both kept at 250 °C. The carrier gas was He at 0.7 mL/min, and the injection was performed in split mode with a 1:10 ratio.

For the analysis of furanic compounds and ethyl esters, the same instrumentation equipment was employed. However, the chromatographic separation occurred on a Supelcowax10 (30 m × 0.53 mm with a 1 μm film thickness, Supelco, Bellefonte, PA, USA) with an initial oven temperature of 30 °C (5 min), then increased to 220 °C at 3 °C/min rate, and held isothermally for 2 min. He gas was at 1 mL/min and the injection was performed in split mode with a 1:10 ratio. The injector and detector were respectively set at 250 °C and 300 °C. 

In both analyses, the identification occurred by direct comparison with the retention times of authentic standards, while the quantification procedure was performed by interpolation of the peak areas in the calibration plots of relative reference compounds followed by an internal standard normalization. Data acquisition and processing were performed by Clarity Chromatography Software v4.0.2 (Dani Instrument, Milan, Italy). Every sample was analyzed in triplicate.

### 2.6. Element Analysis

Approximately 0.5 g of each sample was added with 7 mL of HNO_3_ and H_2_O_2_ and mineralized with a microwave digestion system (Ethos 1, Milestone, Bergamo, Italy) by increasing the temperature from 0–200 °C in 10 min (hold time 10 min), with a microwave power of 1200 W, and subsequent cooling for 20 min. Then, the digested sample was properly diluted with ultrapure water, filtered by means of a 0.45µm pore syringe filter and analyzed by inductively coupled plasma-mass spectrometry (ICP-MS). As already reported in our previous studies, the ICP-MS instrument (iCAP-Q, Thermo Fisher Scientific) was first tuned and the analytical method subsequently optimized for reducing spectral and non-spectral interferences, which could significantly affect the multi-analyte determination [41]. Then, the analysis was performed according to a method reported elsewhere [42,43,44], which included an incident radio frequency power equal to 1500 W and plasma, auxiliary, and carrier gases (argon) at respective flow rates of 15 L/min, 0.9 L/min and 1.10 L/min. The instrument operated in helium collision mode (4 mL/min) and was equipped with a spray chamber set at +2 °C. The injection volume and the sample introduction flow rate were, respectively, 200 μL and 1 mL/min. Spectra acquisition occurred in full scan mode (dwell time 0.5 or 0.01 s/point, depending on the analyte). Data acquisition occurred through Qtegra™ Intelligent Scientific Data Solution™ software (Thermo Scientific, Waltham, MA, USA). For quantification, an external calibration procedure based on suitable six-point calibration curves was carried out for each analyte. Samples were run in triplicate along with analytical blanks.

### 2.7. Sensorial Analysis

A descriptive sensory analysis of the several Amarena wines was conducted according to the standards of the ISO 13299:2016 [45]. A seven member panel (four females and three males between 30 and 60 years old) constituted by technicians, researchers, and professors from the University of Messina with a solid experience in the food and beverage area, including the sensorial analysis of wine, was trained following the criteria of ISO 8586:2012 [46] and the sensory analysis was conducted in a tasting room designed according to the ISO 8589:2007 standard [47]. Specifically, the room had controlled conditions of temperature, humidity, and light and contained 10 tasting booths, each surrounded by walls and ceiling white in color and fitted with a light producing natural day light, a white sink and a frontal white desk allocating the wines to be tested.

According to the ISO 3591:1979 [48], around 30 mL of every Amarena wine was served in an opaque tasting glass that was capped with a watch glass until the imminent sensorial evaluation according to the attributes reported in Table 2. Hence, the panelists were asked to focus on the suggested descriptors and rate the corresponding intensities on an 8-point scale, without any information about the experimental design.

### 2.8. Statistical Analysis

Experimental data were expressed as mean ± standard deviation of *n* = 15 samples *per* wine group, every sample being analyzed three times, and they were visualized by means of Box and Whisker charts.

The effect of bottle aging on the oenological and chromatic properties, volatiles and element profile of Amarena was evaluated by one-way analysis of variance (ANOVA) at *p*-levels of 0.05, 0.01, and 0.001. A Tukey HSD post-hoc test (*p* < 0.05) was then employed to assess significant differences among the different Amarena wines. A Principal Component Analysis (PCA) explored the differentiation of the different Amarena samples based on the relationship between wine composition and bottle aging. PCA is an unsupervised tool that reduces data dimensionality, while identifying those combinations of variables, which provide the largest contribution to sample variability, commonly known as principal components (PCs) [49,50,51,52,53]. To perform PCA, the appropriateness of the data set was first checked by preliminary tests, namely the Kaiser–Meyer–Olkin (KMO) test and the Barlett test. Then, the principal components were extracted using two criteria: examination of the scree plot with eigenvalues >1 and cumulative variance >70% for all the projected data as reported elsewhere [54,55,56]. 

All the statistical procedures were conducted by means of the SPSS 13.0 software package for Windows (SPSS Inc., Chicago, IL, USA).

## 3. Results and Discussion

### 3.1. Oenological Properties

**Figure 1:**
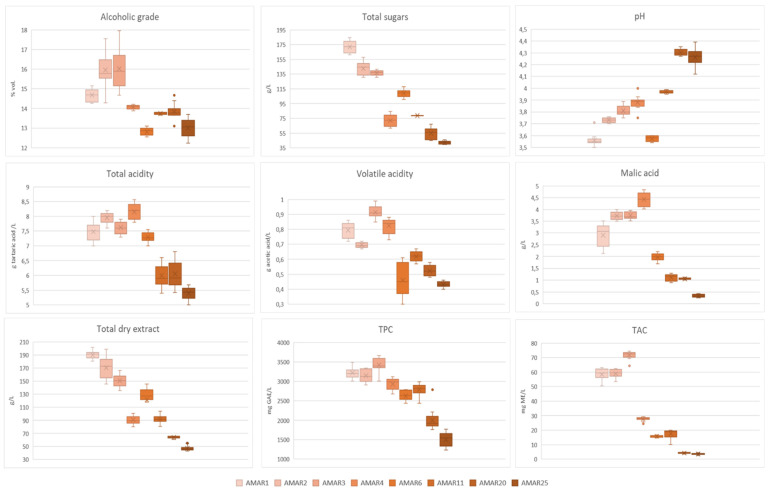
Box and Whisker charts illustrating the oenological traits of Amarena wines in relation to different bottle aging. In each graph, data were expressed as mean ± standard deviation of *n* = 15 samples coming from 5 bottles, where every sample was analyzed three times. The “×” indicates the average value in each wine group, whereas the outlier points display the outlier data lying either below the lower whisker line or above the upper whisker line. TPC: total phenol content; TAC = total anthocyanin content.

The physicochemical data from the various Amarena wines are illustrated in Figure 1 by means of Box and Whisker charts and listed in Table S1. 

Overall, a strict dependence of almost all investigated parameters with the wine aging was pointed out. Specifically, the alcoholic grade tended to increase during the first three years of aging (i.e., AMAR1-AMAR3: 14.68–16.02%, *p* < 0.05) and then decrease up to 25 years (i.e., AMAR4-AMAR25: from 14.06% to 13.11%, *p* < 0.05) (Figure 1 and Table S1). It may be speculated that the reduction of alcoholic strength may be due to the ethanol oxidation in favor of acetaldehyde formation, typically observed during the aging of red wine and fortified wines [57,58].

Downward trends of total dry extract and total sugars could be outlined, as younger Amarena wines had a higher total dry extract (e.g., AMAR1-AMAR3: 190.93–150.01 g/L, *p* > 0.05) and a greater sugar content (e.g., AMAR1-AMAR3: 172.05–136.58 g/L in *p* < 0.05) than older samples (e.g., AMAR20: 63.84 g/L and 55.10 g/L, respectively, and AMAR25: 47.06 g/L and 30.06 g/L, respectively, *p* < 0.05) (Figure 1 and Table S1). Along with the alcoholic degree, the dry extract defines the body of a wine, and it is generally accepted that the more the alcohol and the dry extract the fuller the body of the beverage [54]. Hence, according to the obtained data, younger Amarena wines would be more full-bodied than older ones, due not only to the stronger alcoholic grade and higher levels of residual sugars, but also to greater contents of polyphenols which typically confer a positive perception of density and viscosity in the mouth [59].

Both total acidity and acetic acid significantly increased during the first 4 years of aging (i.e., AMAR1-AMAR4: from 7.48 g/L to 8.15 g/L, *p* > 0.05, and from 0.79 g/L to 0.85 g/L, *p* < 0.05, respectively) and then decreased in Amarena bottles up to 25 years, (i.e., AMAR11-AMAR25: from 5.99 g/L to 5.40 g/L, *p* > 0.05, and from 0.62 g/L to 0.43 g/L, *p* < 0.05, respectively) (Figure 1 and Table S1). The decrease in total acidity observed in older wines may be due to the undesirable precipitation of salts derived from organic acids, such as tartaric acid.

In particular, an acetic acid content exceeding 0.7 g/L would give a sour and pungent aroma to the younger beverages [60], while concentrations between 0.2 and 0.7 g/L would contribute to the complexity of the aroma of older wines [61]. Accordingly, malic acid increased during the first 4 years of wine aging (i.e., AMAR1-AMAR4: from 2.91 g/L to 4.43 g/L, *p* < 0.05) and steadily reduced up to 25 years of aging (i.e., AMAR6-AMAR25: from 1.98 g/L to 0.34 g/L, *p* < 0.05) (Figure 1 and Table S1). This could be due to the malolactic fermentation typically occurring during wine aging. Indeed, the malic acid already present in the wine before the bottling phase does not precipitate like tartaric acid and can initiate a second round of fermentation, by transforming itself into the more delicate and less pungent lactic acid, thus, reducing wine acidity [57,62,63,64]. As expected, the overall reduction of wine acidity corresponded to an increase of the pH wine, as younger Amarena wines generally exhibited lower pH values than the older counterpart (e.g., AMAR1-AMAR4: 3.55–3.88, *p* < 0.05 vs. AMAR20-AMAR25: 4.30–4.26, *p* < 0.05) (Figure 1 and Table S1).

The study of the evolution of phenolic compounds and their main families in relation to winemaking and aging practices represents one of the most important quality parameters of a wine, since they contribute to its organoleptic characteristics, particularly in terms of color and astringency [64,65,66]. The TPC and TAC of Amarena wines showed a similar evolution during the aging in bottle. In fact, wines aged between 1 and 3 years were characterized by the highest TPCs and TACs (i.e., AMAR1-AMAR3, 3221.93–3421.93 mg GAE/L, *p* > 0.05, and 58.50–71.75 mg ME/L, *p* < 0.05). Then, TPC and TAC started to decrease, reaching the lowest values in wines aged up to 25 years (i.e., AMAR20-AMAR25, 1995.73–1501.13 mg GAE/L, *p* < 0.05, and 4.23–3.62 mg ME/L, *p* > 0.05) (Figure 1 and Table S1). In a recent work [36], several productions of a fortified wine based on the sour cherry fruit and recognized as “Vino di Visciole”, a traditional alcoholic beverage from Marche (Italy), exhibited TPCs and TACs in line with this study, as they ranged respectively between 1173.83–2772.96 mg GAE/L and 12–89 mg/L. However, no conclusion could be inferred about the variation of polyphenols in relation to the wine aging.

While recognizing that the characterization of single polyphenols, along with their structure and function becomes necessary in certain frames of the wine production, rapid, simple, and globally accepted spectrophotometric methods are routinely employed to evaluate the phenolic content of a wine, both for research and quality control purposes.

The spectrophotometric methods, such as the well-known Folin–Ciocalteu assay, rely on the concept that the absorbance at 280 nm is characteristic of the benzene ring of most phenolics, except cinnamic acids and chalcones [67]. However, several non-phenolic compounds—such as amino acids, ascorbic acid, reducing sugars, and transition metals [68]—may also absorb at 280 nm, thus, inevitably skewing the real TPC of a wine. This is the main reason why it would sound more scientifically correct to affirm that this assay, rather than determining total polyphenols, measures the reducing properties of a wine, which are directly proportional to its polyphenol content [69]. Another common test based on the direct and fast absorbance measurement of diluted wine at 280 nm and providing the so-called total polyphenol index (TPI), may be interchangeably considered with the Folin–Ciocalteu assay, as it would respond similarly to the variations in the phenolic content of a wine [70].

During the wine aging under non-oxidative conditions (bottle), phenolics typically undergo several condensation reactions [64]. Hence, the changes in TPC observed in Amarena with different bottle aging may be explained by the transformation of phenolic compounds into condensed forms with slightly different chemical properties and reactivities towards the Folin–Ciocalteu reagent. On the other hand, the method used for the assessment of TAC mostly takes into consideration free monomeric anthocyanins, within the large family of polyphenolic compounds [71,72]. Therefore, the downward trend of TAC observed during the aging of Amarena is consistent with the involvement of monomeric anthocyanins in various condensation reactions, and, to a minor extent, with the degradation reactions occurring during the bottle aging [73]. Against this background, although anthocyanins are generally responsible for <4% of the total phenolics in wine, it may be speculated that their decrease may significantly contribute to the concomitant variation of total phenols observed in Amarena wines aged in bottle.

In literature, a variety of well-known fortified wines produced in the Mediterranean area, even labelled by relevant quality marks, such as the Spanish “Fino” Sherry and the Portuguese “Madeira”, were explored in relation to the aging. Although the production and the aging of such wines typically occurs according to strict specifications, the proximity of the traditional Amarena beverage to these fortified wines may be evaluated based on the investigated oenological parameters.

For example, the Portuguese “Madeira” from four different white grape varieties and aged in barrel up to 25 years showed a general increase in the alcoholic degree (from a minimum of 16.8% in younger wines to a maximum of 21.3% in the older ones, depending on the variety) and in total sugars (form a minimum of 49.4 g/L in younger wines to a maximum of 150.8 g/L in the older ones, depending on the variety) with time. Moreover, the pH was kept constant during the whole aging phase [74].

On the other hand, unpublished results from our group on the Sicilian golden “Marsala” aged in wooden barrel for >1 year (“Fine”), >2 years (“Superiore”), >4 years (“Superiore-Riserva”), >5 years (“Vergine”) and >10 years (“Stravecchio”), displayed the same evolution trend for alcoholic grade (from 17.96% to 16.38%), and total dry extract (from 185.20 g/L to 31.25 g/L) over time. However, differently from the Amarena wine, total and volatile acidity increased with wine aging (respectively, 3.37–5.56 g/L and 0.16–0.57 g/L). The Spanish “Fino” sherry aged in barrel up to 5 years according to the *Criaderas y Solera* dynamic system, was most similar to the Sicilian Amarena for the evolution trends of oenological parameters. In fact, this fortified wine was characterized by a slight decrease in the alcoholic strength up to 5 years of aging in barrel (from 16.0% to 15.0%) associated with a simultaneous increase in acetaldehyde levels, and the total and volatile acidity increased during the first/second year of aging (total acidity: from 3.0 g/L to 5.6 g/L; volatile acidity: from 0.29 g/L to 0.50 g/L) and then decreased until 6 years of aging (total acidity: from 5.6 g/L to 4.1 g/L; volatile acidity; from 0.5 g/L to 0.22 g/L). However, differently from what observed in this study, the pH lowered during the wine aging (from 3.51 to 3.22). Similar to the Amarena wine with a few years of aging, the TPI had a growing trend up to 5 years of aging [75].

### 3.2. Chromaticity

**Figure 2:**
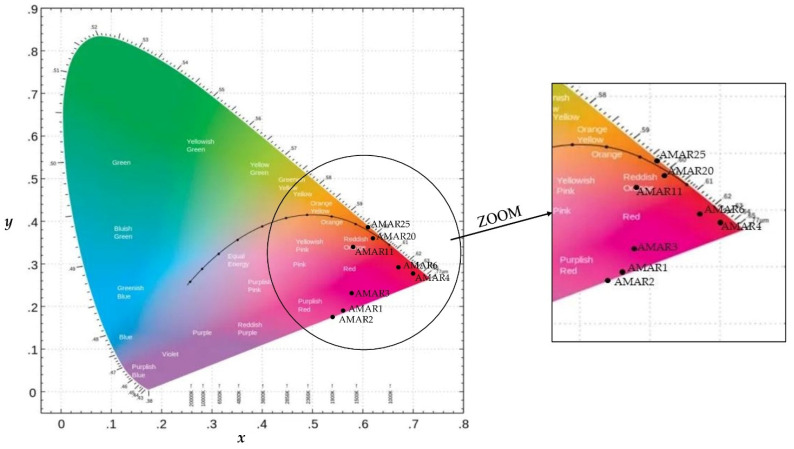
Distribution of the different samples of Amarena wines in the space of the CIE xy chromaticity diagram.

The chromaticity of the different Amarena wines is illustrated in Figure 2. In Table S2, the coordinates of luminosity (Y), hue (x) and purity (y) responsible for the chromaticity of every sample are also listed.

Overall, younger AMAR1-AMAR3 wines were characterized by a purplish-red hue (i.e., x: 0.56–0.58, *p* > 0.05, y: 0.19–0.23, *p* > 0.05) and a scarce luminosity (i.e., Y: 3.53–3.38, *p* > 0.05). With advancing aging, the wines AMAR4 and AMAR6 had a slightly increased luminosity (i.e., Y: 3.45–5.18, *p* < 0.05) and the purple notes turned to an intense red hue as well (i.e., x: 0.70–0.67, *p* > 0.05, y: 0.28–0.29, *p* > 0.05). Finally, the oldest AMAR11, AMAR20 and AMAR25 exhibited a greater transparency and brightness as well as a marked brownish-red hue (i.e., Y: 14.44–17.60, *p* < 0.05, x = 0.58–0.61, *p* > 0.05; y = 0.34–0.39, *p* > 0.05) (Table S2 and Figure 2).

The color of a wine is the first attribute assessed by consumers during the tasting and, thus, it is one of the most important characteristics in the construction of the acceptability of the product [76]. Indeed, color provides information about the type of wine, winemaking or aging process and can often anticipate the taste and/or odor properties based on the previous experience of the consumer [77].

Anthocyanins are the main polyphenols responsible for the color of a wine. These molecules are relatively water-soluble, exhibit color thanks to the resonant structure of their flavylium ion [78,79,80], and are usually linked to a glucose molecule, which can be hydrolyzed, thus, allowing the release of less stable anthocyanidins [81,82], such as cyanidin, delphinidin, peonidin, petunidin and malvidin, very common in grape skins. Anthocyanins are gradually extracted from skins during the early stages of winemaking and provide musts and young wines their typical bluish-red color. Subsequently, condensation reactions of the anthocyanin chromophores with flavan-3-ols (copigmentation) [83,84] or with other anthocyanin (autoassociation), whether direct or mediated by acetaldehyde—mainly coming from ethanol oxidation—allow the conversion of anthocyanins into oligomeric and polymeric pigments. Under the right oxidation conditions, these more stable pigments stabilize the wine color, providing typical purplish-red notes by the first year of aging [85]. During the bottle storage, spontaneous clearing, color stabilization and further reactions takes place. Among others, cyclo-addition reactions between acetaldehyde and anthocyanins, as well as condensation reactions between anthocyanins and other compounds of lower molecular weight (i.e., pyruvic acid, vinylphenol or glyoxylic acid) lead to the formation of the so called pyranoanthocyanins, responsible for brownish red notes, typical of older wines [64,86,87].

In view of this, the change observed in the chromaticity of Amarena wines may be explained by the concomitant variation of anthocyanins. In fact, as already observed in Table 1, free monomeric pigments of anthocyanins, responsible for a purple-red color, decreased during the wine aging because they were likely displaced by more stable polymeric pigments, responsible for orange-red hues [81,88].

Accordingly, the literature has already pointed out that the decrease of the concentration of free anthocyanins in favor of the formation of polymeric pigments, causes a definite evolution in the wine color. The purple-red color of young Port wines, for example, typically turns towards yellow-brown color with aging [89]. In accordance with the results from this study, Spanish red sweet wines exhibited increasing hue values during bottle storage and a color evolution toward orangish notes, due to the formation of stable pigments at the expense of monomeric anthocyanins [90].

### 3.3. Element Analysis

Data on major and trace element from Amarena wines with diverse bottle aging are illustrated in Figure 1 by means of Box and Whisker charts and listed in Table S3.

**Figure 3:**
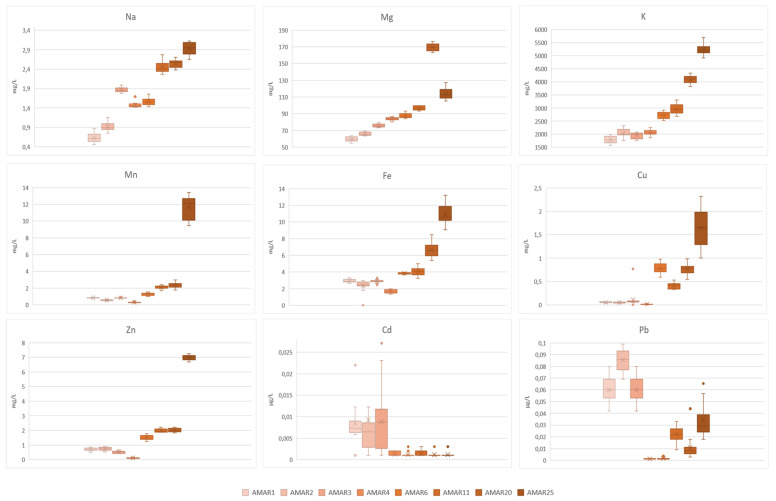
Box and Whisker charts illustrating the levels of major and trace elements of Amarena wines in relation to different bottle aging. In each graph, data were expressed as mean ± standard deviation of *n* = 15 samples coming from 5 bottles, where every sample was analyzed three times. The “×” indicates the average value in each wine group; whereas the outlier points display the outlier data that lie either below the lower whisker line or above the upper whisker line.

Level of minerals, such as Na, Mg and K, tended to increase with the advancement of the bottle aging of wines. K and Mg were the most abundant elements, with a content varying over the investigated aging period from 59.59 mg/L to 113.78 mg/L (*p* < 0.05) and from 1784.03 mg/L to 5225.35 mg/L (*p* < 0.05), respectively (Figure 3 and Table S3). Particularly, K is strongly influenced by the precipitation of potassium bitartrate in the early stages of aging, mainly due to the oversaturation of this salt, its relatively low solubility in the hydroalcoholic medium, and the low temperatures of the cellar. In turn, the oversaturation of potassium in the wine could be partly responsible for the increase of pH observed in older Amarena wines (Figure 1) [91].

Similarly, trace essential elements, such as Mn, Fe, Cu, and Zn showed a growing trend up to 25 years of wine aging in bottle. Interestingly, older wines displayed levels of Cu and Zn (i.e., AMAR25-AMAR11: 1.64–0.41 mg/L of Cu, *p* < 0.05; and 7.00–1.99 mg/L of Zn, *p* < 0.05) respectively 30 and 10 orders of magnitude higher than younger Amarena bottles (i.e., AMAR4-AMAR1: 0.015–0.052 mg/L of Cu, *p* < 0.05; and 0.11–0.69 mg/L of Zn, *p* < 0.05) (Figure 3 and Table S3).

However, potentially toxic trace elements, such as Cd and Pb, had an opposite evolution during the wine aging. Specifically, Cd evolved steadily in wines aged for 25 and 4 years (i.e., AMAR25-AMAR4: 1.17–1.27 µg/L, *p* > 0.05), and then it sharply increased in younger wines (i.e., AMAR3-AMAR1: 8.88–8.43 µg/L, *p* > 0.05). Pb was found at quite high levels in the older Amarena bottles (i.e., AMAR25-AMAR11: 34.3–22.27 µg/L, *p* > 0.05), then it significantly reduced in wines with intermediate aging periods (i.e., AMAR6-AMAR4: 1.65–1.25 µg/L, *p* > 0.05) and, finally, it markedly increased in younger Amarena bottles (i.e., AMAR3-AMAR1: 57.65–60.07 µg/L, *p* > 0.05). The relevant contents of Pb and Cd in older Amarena samples may reflect the progressive deterioration of the surrounding environment in terms of anthropogenic pollution.

However, all Amarena wines were demonstrated to be safe for the human consumption because their Pb content was well within the regulatory limits set by Reg. (CE) 1881/2006 for this heavy metal (0.2 mg/Kg) [92]. Additionally, the element profile of most of the investigated wines was in accordance with the limits of certain elements contained in wines set by the National Decree-Law of August 10, 2017 [93], except for the oldest Amarena, which showed Cu and Zn contents slightly above the regulatory thresholds (respectively, 1 mg/L and 5 mg/L).

A literature overview pointed out that the study of the element profile of a wine has been conducted mainly for botanical or geographical traceability purposes, as certain inorganic elements are strictly related to the grape variety or the composition of the soil in which the vineyard grows [94,95,96]. As a result, few studies focused on the exploration of the element fingerprints of a red wine in relation to the aging practice [97,98,99], and even fewer studies considered the fortified wines [100,101].

Overall, these studies highlighted mean contents of most elements of wine consistent with the level of minerals and trace metals described for the Amarena wine, and, interestingly, the aging process confirmed to cause an overall increase of these inorganic elements. In the Golden Marsala wines aged in oak barrels and refined in bottles, for example, Zn ranged from 0.4 mg/L to 3.1 mg/L, Cu increased from 0.8 mg/L to 0.9 mg/L, Cd varied between 2.2–120 µg/L, and Pb increased from 18.7 µg/L to 130 µg/L, when considering wine aged from 12 (Marsala “Fine”) to 132 (Marsala “Stravecchio”) months [100].

Hence, the aging process of a wine may significantly affect the solid relationship between wine-grape variety or wine-soil, and, as such, it deserves to be further explored also in relation to the element profile.

### 3.4. Volatiles

**Figure 4:**
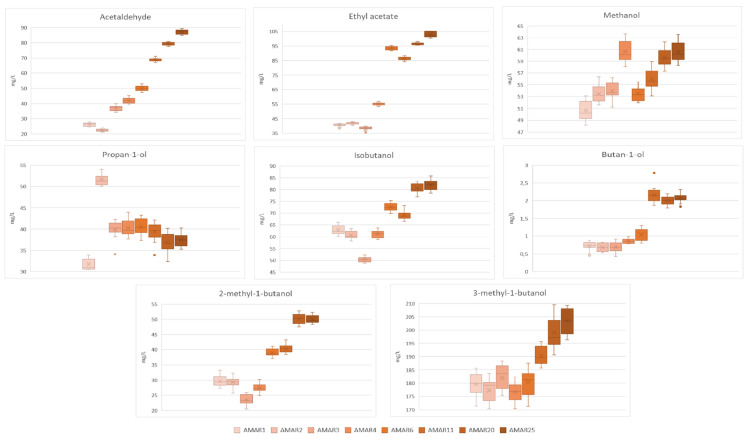
Box and Whisker charts illustrating the levels of acetaldehyde, ethyl acetate, methanol and higher alcohols of Amarena wines in relation to different bottle aging. In each graph, data were expressed as mean ± standard deviation of *n* = 15 samples coming from 5 bottles, where every sample was analyzed three times. The “×” indicates the average value in each wine group; whereas the outlier points display the outlier data that lie either below the lower whisker line or above the upper whisker line.

**Figure 5:**
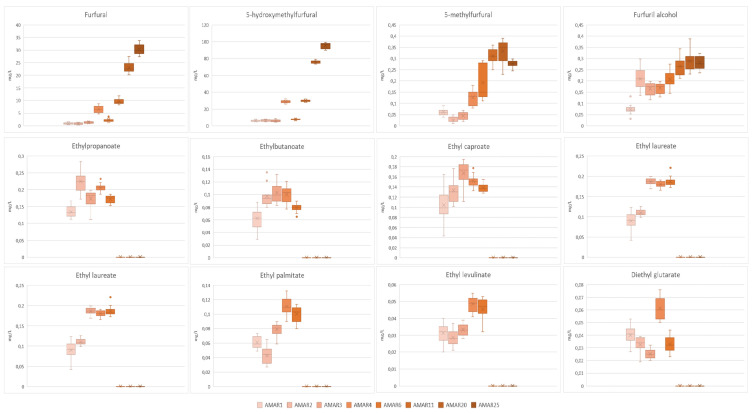
Box and Whisker charts illustrating the levels of furanic compounds and ethyl esters of Amarena wines in relation to different bottle aging. In each graph, data were expressed as mean ± standard deviation of *n* = 15 samples coming from 5 bottles, where every sample was analyzed three times. The “×” indicates the average value in each wine group; whereas the outlier points display the outlier data that lie either below the lower whisker line or above the upper whisker line.

The contents of volatile compounds of the Amarena wines with different bottle ages are illustrated in Figure 4 and Figure 5 and listed in Table S4.

Notoriously, acetaldehyde, ethylacetate and methanol are the major markers of wine aging, as acetaldehyde derives from the ethanol oxidation and yeast metabolism during and after alcohol fermentation as well [102]; ethyl acetate is obtained from yeast and acetic bacteria metabolism; while methanol is derived from enzymatic degradation of grape pectins in the fruits and during the alcoholic fermentation [103,104]. Except for methanol, which is not involved in the flavor, aroma and mouth-feel of wine [104], the acetaldehyde may enhance the fruity aroma at a low concentration. However, at higher levels, it is reminiscent of ethereal and ripe apple notes, and above 100 mg/L is then considered as a defect [105]. Also, the condensation reactions between acetaldehyde and phenolics during aging may reduce the astringency of the wine [106]. Ethyl acetate may impart favorable notes on wine aroma at levels lower than 80 mg/L. However, in aged wines it is responsible for the typical attribute of acescency [107]. In the present work, these three volatiles followed a peculiar evolution during the aging of Amarena wine, thus, confirming the advancement of an oxidation process which takes place most significantly during barrel aging and, ultimately, bottle storage, as along as the whole aroma of wine is altered [108]. Indeed, the acetaldehyde grew over the years ranging from 26.19–50.01 mg/L (*p* < 0.05) in younger wines (AMAR1-AMAR6) to 68.89–87.17 mg/L (*p* < 0.05) in older beverages (AMAR11-AMAR25). Similarly, ethyl acetate and methanol increased with advancing bottle aging, as they varied respectively between 40.56–102.87 mg/L (*p* < 0.05) and 50.69–60.51 mg/L (*p* < 0.05) (Figure 4 and Table S4).

In literature, other renowned fortified wines were generally marked by increasing levels of acetaldehyde, ethylacetate and methanol during maturation. “Fino” sherry wines displayed increasing trends for acetaldehyde (91–257 mg/L), ethyl acetate (66.7–85.5 mg/L) and a decreasing content of methanol (80.1–68.6 mg/L) during five years of biological aging [109]. Madeira wines from the varieties Boal, Malvazia, Sercial and Verdelho had more similar contents of such compounds with respect to the Amarena wines, as growing levels of acetaldehyde (respectively, 20.62–116.99 mg/L, 18.38–57.82 mg/L, 27.19–85.78 mg/L, and 15.60–81.48 mg/L), ethyl acetate (respectively, 24.01–264.36 mg/L, 30.22–101.54 mg/L, 2.98–156.24 mg/L, and 26.56–125.70 mg/L), and methanol (respectively, 51.00–88.46 mg/L, 86.57–88.44 mg/L, 57.18–152.85 mg/L, and 73.95–103.40 mg/L) were highlighted up to 25 years of oxidative aging [74].

Higher alcohols, also known as fusel alcohols, are products of yeast fermentation and can derive both from carbohydrates by the Embden–Meyerhof–Parnas pathway and amino acids via the Ehrlic pathway [110]. Along with the relative esters, they play an important role in wine aroma. In fact, a low total concentration of these alcohols (~300 mg/L) positively contributes to the aromatic complexity of wine, whereas a higher level may provide “solvent” or “fuel” odor. In this respect, propanol is marked by descriptors such as alcohol and ripe fruit; isobutanol alcohol, wine-like aroma descriptors and nail polish; and isoamyl alcohols (i.e., 2-methyl-1-butanol and 3-methyl-1-butanol) give notes such as nail polish and fuel alcohols [111,112].

In this study, higher alcohols, including propan-1-ol, isobutanol, butan-1-ol, 2-methyl-1-butanol and 3-methyl-1-butanol, were successfully identified in all samples by means of authentic standards. The quantitative analysis pointed out that, with the exception of propan-1-ol which exhibited a quite steady content over time (31.73–37.52 mg/L, *p* < 0.05), the other higher alcohols becoming higher with the bottle aging perhaps as a consequence of hydrolysis of the relative esters or evaporation phenomena [74]. Among them, in particular, isobutanol and 3-methyl-1-butanol were found at the highest content (respectively, 62.89–81.82 mg/L, *p* < 0.05, and 179.67–203.35, *p* < 0.05). Additionally, non-significant differences were observed in the level of total higher alcohols of the Amarena wines aged for 1–4 years, which showed values ranging between 304.64 and 306.57 mg/L (*p* > 0.05), thus, being around the threshold of sensory acceptability. However, according to the ANOVA test, younger Amarena were significantly different from the fortified wines aged over 11–25 years, which had a higher total content, varying between 341.42–374.75 (*p* > 0.05), indicative of potentially perceptible sensory defects (Figure 4 and Table S4). Higher alcohols contents found in the Amarena wines were similar to those obtained for Madeira wines from the variety Sercial aged under oxidative conditions over 1–25 years (259.38–372.91 mg/L). However, the same fortified wines produced from different grape varieties exhibited much lower levels of total higher alcohols (i.e., Boal: 39.42–293.60 mg/L, Malvazia: 96.72–149.04 mg/L, and Verdelho: 58.89–221.07 mg/L) [74]. On the other hand, “Fino” sherry wines showed higher levels of propan-1-ol (34.8–71.5 mg/L) and isoamyl alcohols (300–380 mg/L), and a slightly lower content of isobutanol (48.0–70.6 mg/L) over 5 years of biological aging [109].

Furanic compounds not only contribute significantly to the complexity of wine aroma. Indeed, predominant furans of aged wines—i.e., 2-furfural, 5-methyl-2-furfural, and 5-hydroxymethyl-2-furfural (HMF)—have been associated with aromatic notes of caramel, spice, brown, and dried fruits [109,113,114,115]. They are the main degradation products of carbohydrates and their occurrence in food is generally due to non-enzymatic browning reactions, namely sugar degradation in acid medium, Maillard reaction, and caramelization. In wine, however, the Maillard and caramelization chemistry are not favored due to the low pH of the medium, and, consequently, these compounds are obtained by the acid-catalyzed sugar degradation [116]. Furanic derivatives in wine may derive also by the degradation of carbohydrates during the toasting of the barrel. In this respect, the quantity of such volatiles in wine is affected by the age of the barrel, the degree of wood toasting, and not least, the time period of wine maturation in the barrel [74,117]. Amarena wines were characterized by furanic compounds, such as 2-furfural, HMF, 5-methylfurfural, and furfuril alcohol, increasing over the bottle aging. Furfural and HMF were the most abundant volatiles varying respectively between 0.89–30.35 mg/L and 6.06–95.54 mg/L in Amarena samples. On the other hand, 5-methylfurfural and furfuril alcohol were present in lower and similar contents, ranging respectively from 0.062 and 0.07 mg/L to 0.27 and 0.28 mg/L (Figure 5 and Table S4).

A literature overview pointed out that, although highly variable, furanic compounds, such as 2-furfural and HMF, could be considered as ageing markers of fortified wines. Madeira wines from different varieties (i.e., Boal, Malvazia, Sercial and Verdelho) and matured in oak barrels during 1–25 years were characterized by contents quite similar to those of older Amarena wines. In fact, aromas such as 2-furfural and HMF varied respectively within the range 0.2–24.1 mg/L and 0.7–100.3 mg/L [74]. On the other hand, “Fino” sherry wines subject to biological maturation over 5 years had a content of 2-furfural comparable to that of Amarena wines with similar aging times, as it was comprised of between n.d.-4.8 mg/L. Nevertheless, these wines developed higher levels of 5-methylfurfural and furfuril alcohol (i.e., n.d.–0.86 mg/L and 0.110–0.556 mg/L) [109]. Ortu and Carboni [118] screened the furanic derivates of different types of Marsala and they pointed out that different samples of “Fine” semi-dry amber Marsala- with minimum 1 year of oxidative ageing- were marked by a content of 2-furfural and HMF lower than “Superiore” dry amber Marsala- with minimum 4 years of oxidative ageing (2-furfural: 2.0–5.0 mg/L vs. 5.5 mg/L; HMF: 97–140 mg/L vs. 511 mg/L).

Ethyl esters of straight medium-chain fatty acids (C6–C12) are typical products of lipid yeast metabolism thanks to reactions between ethanol and acyl-coenzyme A derivatives, whose content depends on variables such as yeast strain, fermentation conditions, degree of aeration during maturation, and sugar levels in wine. Of particular relevance are ethyl propanoate, ethyl 2-methylpropanoate, and ethyl 2-methylbutanoate which provide blackberry aromas, and ethyl butanoate, ethyl caproate, ethyl caprylate, and ethyl caprate, which typically exhibit red fruity odors, thus, positively contributing to the wine aroma [74,119].

Different ethyl esters, including ethyl propanoate, ethyl butanoate ethyl caproate, ethyl laureate, ethyl myristate, ethyl palmitate, ethyl levulinate and diethyl glutarate, were successfully identified and quantified by means of authentic standards only in AMAR1-AMAR6 samples, thus, resulting not detected in the older wines AMAR11-AMAR25. Indeed, such volatiles are generally hydrolyzed during wine aging, their level being particularly affected by low pH and high temperature of the medium as well as by the equilibrium between the esters and the corresponding acids [120,121,122]. However, ethyl esters mostly kept a constant content in young Amarena wines, with the most abundant ethyl myristate (0.19–0.24 mg/L, *p* > 0.05), ethyl propanoate (0.13–0.22 mg/L, *p* > 0.05) and ethyl caproate (0.10–0.19 mg/L, *p* < 0.05), and the least concentrated ethyl levulinate (0.029–0.048 mg/L, *p* < 0.05) and diethyl glutarate (0.025–0.061 mg/L, *p* < 0.05) (Figure 5 and Table S4).

A literature comparison showed that “Fino” sherry wines biologically aged up to 5 years reported growing levels of ethyl propanoate (0.091–1.29 mg/L), ethyl butanoate (0.161–1.49 mg/L), ethyl caproate (0.092–0.176 mg/L) [109]. Conversely, the total amount of fatty acids ethyl esters unequivocally decreased by ~45% in the first 11 years of maturation in oak barrels, and by ~25% during the second 14 years of aging, in Madeira wines from different grape varieties [74].

### 3.5. Differentiation of Amarena Wines by PCA

In this study, PCA was run for testing the capacity of oenological parameters, chromatic properties, inorganic elements, and volatile compounds to differentiate Amarena wines according to the aging.

Coherently with the Kaiser criterion, only the first four PCs with eigenvalues exceeding 1 were extracted from the scree plot. They accounted for a total variance of 83.88%.

**Figure 6:**
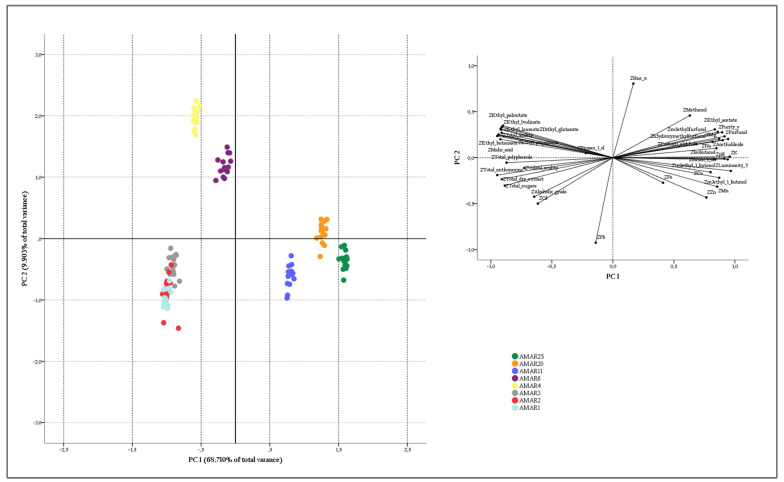
Bidimensional score (**a**) and loading (**b**) plots of investigated physico-chemical parameters over the Amarena wine with different aging. Drawn ellipses suggest the natural grouping of samples (*n* = 15 samples per wine group) according to the aging period.

The score and loading plots for the first two PCs explaining 78.61% of the total variance is illustrated in Figure 6. Considering the score plot, Amarena wines were not completely differentiated according to the age in the component space. In fact, although most of samples (AMAR4-AMAR25) formed definite clusters on PC1, explaining 68.71% of the total variance, samples from younger Amarena wines (AMAR1-AMAR3), completely overlapped on PC1, explained only 9.90% of the total variance (Figure 6a). As a result, it may be argued that the differentiation of Amarena wines occurred more consistently with the increase in aging. On the other hand, the loading plot revealed a clear clustering among elements (i.e., Mg, Na, Mn, Zn, and Cu), acetaldehyde, methanol, ethyl acetate, higher alcohols, furanic compounds, chromatic properties (i.e., luminosity and purity), and the pH, which were characterized by the highest positive loading values on PC1. A strong association among all the other oenological parameters and ethyl esters was also present, as they exhibited the highest negative loading values on PC2. However, variables such as Pb, Cd, Fe and hue, respectively, with lower negative and positive loading values, were more dispersed into the component space, suggesting an individualized behavior (Figure 6b).

By overlapping the loading and score plots, it was evident that elements, such as Mg, Na, Mn, Zn, and Cu, most of the volatiles investigated, and chromatic properties, such as luminosity and purity and pH, were useful for differentiating the oldest Amarena wine from the other Amarena bottles, since such parameters were revealed at the highest levels in the AMAR11-AMAR25 samples. Interestingly, the hue differentiated young Amarena wines (AMAR4-AMAR6) from wines with relevant aging periods (AMAR11-AMAR25), due to a deep red hue that turned toward characteristic brownish-red notes of older products. On the other hand, the various oenological parameters and ethyl esters were plotted against younger wines (AMAR3-AMAR1), thus, demonstrating a scarce discriminating power towards such samples.

### 3.6. Sensorial Analysis

**Figure 7:**
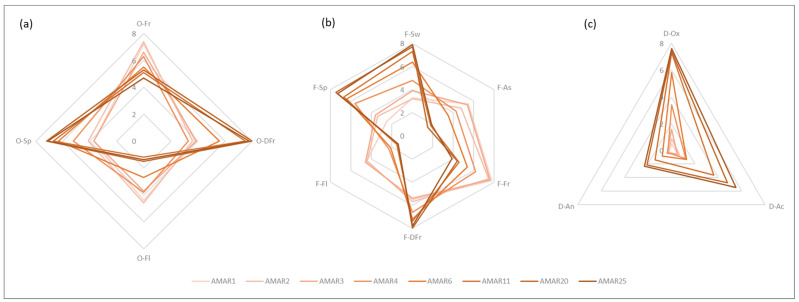
Sensorial analysis of investigated Amarena wines with different aging conducted in terms of odor (**a**), flavor (**b**) and defects (**c**). Scores were based on a 0–8-point numeric scale and they were reported as mean ± standard deviation (*n* = 7). For the odor: O-Fr, Red fruits; O-DFr, Dried fruits; O-Fl, Floral; O-Sp, Spicy. For the flavor: F-Sw, Sweetness; F-As, Astringency; F-Fr, Red fruits; F-DFr, Dried fruits; F-Fl, Floral; F-Sp, Spicy. For the defects: D-O, Oxidized; D-Ac, Acidity; D-An, Animal.

Figure 7 reports the outcome of the sensorial analysis carried out on the Amarena wines with different aging. In this respect, the descriptors were catalogued based on the odor, the flavor, and defects (intended as both odor and flavor).

Concerning the odor, younger wines were characterized by fruity and floral aromas more pronounced then older Amarena bottles (i.e., AMAR1-AMAR4: 7.2–6.3, *p* > 0.05 vs. AMAR6-AMAR25: 5.5–4.7, *p* > 0.05). Particularly, red fruits and prune notes were well differentiated. However, with the advance of aging these notes left room for spicy traits (i.e., AMAR1-AMAR4: 3.5–5.2, *p* < 0.05 vs. AMAR6-AMAR25: 6.3–7.1, *p* > 0.05), and dried fruits attributes (i.e., AMAR1-AMAR4: 3.8–3.3, *p* > 0.05 vs. AMAR6-AMAR25: 5.6–7.8, *p* < 0.05).

On the palate, descriptors such as sweetness and astringency exhibited an opposite trend, since sweetness increased (i.e., AMAR1-AMAR25: 3.3–7.9, *p* < 0.05) while astringency decreased (i.e., AMAR1-AMAR25: 5.5–1.9, *p* < 0.05) over time. On the other hand, the other flavor notes had a similar behavior to the corresponding odor attributes. As a result, red fruits, and floral attributes were more marked in younger beverages than the older counterpart (i.e., AMAR1-AMAR4: 7.8–6.2, *p* > 0.05 vs. AMAR6-AMAR25: 5.4–3.9, *p* > 0.05); while notes of dried fruits, such as dried fig and nut, and spices, such as black pepper and nutmeg, become higher as the bottle ages (i.e., AMAR1-AMAR4: 5.5–6.6, *p* < 0.05 vs. AMAR6-AMAR25: 7.2–7.8, *p* > 0.05).

Overall, these findings were in line with previous literature reporting that wine ageing mainly causes the loss of fruit and floral aromas, characteristic of young wines, as well as the production of new aromas, such as “dry fruity”, “nutty” and ‘spicy’, which are derived from storage in a barrel, and kept intensifying over time [123,124].

Considering the defects, the valuable note of “oxidized” appeared with age, making the most aged Amarena bottles similar to the Sherry wine (i.e., AMAR11-AMAR25: 7.3–7.6, *p* > 0.05). Moreover, such wines were also characterized by less valuable defects, such as attributes of acidity and leather, resulting, however, in being slightly perceptible to both the smell and the palate (i.e., AMAR11-AMAR25: 3.63–5.5, *p* < 0.05, and 1.4–2.3, *p* > 0.05, respectively).

## 4. Conclusions

In this study, the traditional alcoholic beverage Amarena, produced in Sicily (Italy) and marked by the TAP quality logo, was investigated for various physico-chemical properties, volatiles, chromatic and sensorial traits in relation to different aging periods.

Notwithstanding that the oenological parameters followed peculiar evolution trends already explained in the literature in the context of fortified wines, the statistical analysis showed that most of such parameters, along with ethyl esters, had a scarce discriminating power only towards younger wines. Conversely, the pH of these wines, certain inorganic elements (i.e., Mg, Na, Mn, Zn, and Cu) and most of the volatiles investigated (i.e., acetaldehyde, methanol, ethyl acetate, higher alcohols and higher alcohols) proved to be key variables in the sample differentiation, especially when considering the older Amarena samples.

Interestingly, the chromaticity traits of such fortified wines, studied in terms of luminosity, hue and purity, were highly responsive to the aging process, since young fortified red wines were typically characterized by a purplish-red color which turned towards a brownish-red hue with aging.

In the broader perspective of the valorization of traditional beverages, the scientific characterization of Amarena wines conducted in this study may encourage its production and commercialization, thus preserving the traditional ecological knowledge and cultural diversity of the Mediterranean area.

## Figures and Tables

**Table 1:** Amarena wines and relative ages determined at the time of bottle opening and sampling (January 2022). For every production, *n* = 5 bottles were considered and *n* = 15 samples were collected.

No.	Code	Age (Year)	*n* Bottles (Samples)
1	AMAR1	1	5 (15)
2	AMAR2	2	5 (15)
3	AMAR3	3	5 (15)
4	AMAR4	4	5 (15)
5	AMAR6	6	5 (15)
6	AMAR11	11	5 (15)
7	AMAR20	20	5 (15)
8	AMAR25	25	5 (15)

**Table 2:** Various descriptors and relative codes employed for the sensorial analysis of Amarena wines.

	Attribute	Code
Odor	Red fruits	O-RFr
	Dried fruits	O-DFr
	Floral	O-Fl
	Spicy	O-Sp
Flavor	Sweetness	F-Sw
	Astringency	F-As
	Red fruits	F-RFr
	Dried fruits	F-DFr
	Floral	F-Fl
	Spicy	F-Sp
Defects (odor + flavor)	Oxidized	D-Ox
	Acidity	D-Ac
	Animal	D-An

## Data Availability

Data is contained within the article or Supplementary Material.

## Supplementary Materials

The following supporting information can be downloaded at: https://www.mdpi.com/article/10.3390/foods11142152/s1, Table S1. Oenological properties of investigated Amarena wines in relation to different bottle aging. For every parameter, data were expressed as mean ± standard deviation of *n* = 15 samples coming from five bottles, where every sample was analyzed three times. TPC: total phenol content; TAC = total anthocyanin content; Table S2. Chromaticity of samples from Amarena wines in relation to different bottle aging. For every parameter, data were expressed as mean ± standard deviation of *n* = 15 samples coming from five bottles, where every sample was analyzed three times; Table S3. Element profile of Amarena wines in relation to different bottle aging. Data were expressed as mean ± standard deviation of *n* = 15 samples coming from five bottles, where every sample was analyzed three times. Table S4. Volatile compounds (mg/L) of Amarena wines during bottle aging. Data were expressed as mean ± standard deviation of *n* =15 samples coming from 5 bottles, where every sample was analyzed three times.
