# Supplementary material for: Valorization of Traditional Alcoholic Beverages: The Study of the Sicilian Amarena Wine during Bottle Aging
Source: Foods. 2022 Jul 20;11(14):2152. doi: 10.3390/foods11142152 (PMC9322394; doi:10.3390/foods11142152)
Supplement: Supplementary file 1 [file foods-11-02152-s001.zip › foods-1813265-supplementary.pdf]

Supplementary Material

Table S1. Oenological properties of investigated Amarena wines in relation to bottle aging. For every parameter, data were expressed as mean ± standard deviation of n=15 samples coming from 5 bottles, where every sample was analyzed three times. TPC: total phenol content; TAC= total anthocyanin content.

Sample	Alcoholic grade (% vol.)	pH	Total dry extract (g/L)	Total sugars (g/L)	Total acidity (g/L tartaric acid)	Volatile acidity (g/L acetic acid)	Malic acid (g/L)	TPC (mg GAE/L)	TAC (mg ME/L)
**AMAR1**	14.68±0.30^{a}	3.56±0.05^{a}	190.93±6.11^{a}	172.05±8.25^{a}	7.48±0.30^{a}	0.79±0.05^{a}	2.91±0.46^{a}	3221.93±141.74^{a}	58.50±3.75^{a}
**AMAR2**	15.96±0.91^{b}	3.73±0.02^{b}	170.47±16.04^{a,b}	142.68±8.51^{b}	7.93±0.22^{a,b}	0.70±0.02^{b}	3.73±0.18^{b}	3147.47±157.05^{a}	59.02±3.05^{a}
**AMAR3**	16.02±0.98^{b}	3.81±0.04^{c}	150.74±9.48^{b}	136.58±9.55^{b}	7.62±0.20^{a,b}	0.91±0.04^{c}	3.74±0.15^{b}	3421.93±185.09^{a}	71.75±2.57^{b}
**AMAR4**	14.06±0.11^{c}	3.88±0.05^{c}	90.84±6.66^{c}	72.05±4.25^{c}	8.15±0.26^{b}	0.82±0.05^{a}	4.43±0.29^{c}	2927.33±140.24^{b}	27.66±1.54^{c}
**AMAR6**	12.82±0.18^{d}	3.57±0.02^{a}	129.93±8.59^{d}	108.99±8.77^{d}	7.29±0.17^{a}	0.46±0.02^{d,e}	1.98±0.17^{d}	2623.87±123.15^{b}	15.77±0.71^{d}
**AMAR11**	13.85±0.36^{e}	3.97±0.01^{d}	111.77±6.08^{e}	91.80±6.35^{c}	5.99±0.37^{c}	0.62±0.03^{f}	1.12±0.14^{e}	2792.00±144.05^{b}	17.71±2.91^{d}
**AMAR20**	13.74±0.26^{c,e}	4.30±0.03^{e}	63.84±1.58^{f}	55.10±2.79^{e}	6.06±0.45^{c}	0.52±0.04^{d}	1.06±0.03^{e}	1995.73±259.84^{c}	4.23±0.49^{e}
**AMAR25**	13.11±0.45^{d}	4.26±0.07^{f}	47.06±3.66^{g}	30.06±2.28^{f}	5.40±0.21^{c}	0.43±0.02^{d,e}	0.34±0.06^{f}	1501.13±167.76^{d}	3.62±0.55^{e}

a–g: different superscript letters in the same column indicate significantly different values for a given parameter ($p < 0.05$ by post hoc Tukey's HSD test); same superscript letters in the same column indicate not significantly different values for a given parameter ($p > 0.05$ by post hoc Tukey's HSD test).

Table S2. Chromaticity of samples from Amarena wines in relation to bottle aging. For every parameter, data were expressed as mean ± standard deviation of n=15 samples coming from 5 bottles, where every sample was analyzed three times.

Sample	Luminosity (Y)	Hue (x)	Purity (y)
**AMAR1**	3.53±0.30[a]	0.56±0.02[a]	0.19±0.03[a]
**AMAR2**	3.21±0.24[a]	0.54±0.05[a]	0.18±0.02[a]
**AMAR3**	3.38±0.58[a]	0.58±0.04[a]	0.23±0.03[a,b]
**AMAR4**	3.45±0.31[a]	0.70±0.05[b]	0.28±0.03[b]
**AMAR6**	5.18±0.33[b]	0.67±0.04[b,a]	0.29±0.04[b,c]
**AMAR11**	14.44±0.65[c]	0.58±0.04[b,a]	0.34±0.02[c]
**AMAR20**	13.33±0.67[c]	0.62±0.04[b,a]	0.36±0.03[c]
**AMAR25**	17.60±0.26[d]	0.61±0.03[b,a]	0.39±0.03[c]

a–g: different superscript letters in the same column indicate significantly different values for a given parameter ($p < 0.05$ by post hoc Tukey's HSD test); same superscript letters in the same column indicate not significantly different values for a given parameter ($p > 0.05$ by post hoc Tukey's HSD test).

Table S3. Element profile of Amarena wines in relation to bottle aging. Data were expressed as mean ± standard deviation of n=15 samples coming from 5 bottles, where every sample was analyzed three times.

Sample	Na (mg/L)	Mg (mg/L)	K (mg/L)	Mn (mg/L)	Fe (mg/L)	Cu (mg/L)	Zn (mg/L)	Cd (μg/L)	Pb (μg/L)
**AMAR1**	0.63±0.12[a]	59.59±4.88[a]	1784.03±237.42[a]	0.85±0.03[a]	2.75±0.35[a]	0.052±0.012[a]	0.69±0.11[a]	8.43±2.53[a]	60.07±8.76[a]
**AMAR2**	0.92±0.06[b]	66.12±1.97[a]	2045.67±109.27[a]	0.57±0.02[b]	2.53±0.18[a]	0.049±0.018[a]	0.72±0.09[a]	9.30±3.77[a]	85.60±10.75[b]
**AMAR3**	1.87±0.13[c]	75.81±1.95[b]	1959.00±162.00[a]	0.83±0.06[a]	2.90±0.35[a]	0.12±0.012[b]	0.52±0.12[a]	8.88±1.48[a]	57.65±9.86[a]
**AMAR4**	1.48±0.07[d]	84.03±1.84[c]	2067.88±112.59[a]	0.30±0.04[c]	1.68±0.21[b]	0.015±0.007[d]	0.11±0.03[b]	1.27±0.42[b]	1.25±0.33[c]
**AMAR6**	1.57±0.09[d]	87.75±2.96[c]	2724.22±120.63[b]	1.27±0.15[d]	3.85±0.09[c]	0.79±0.10[e]	1.52±0.16[c]	1.20±0.41[b]	1.65±0.56[c]
**AMAR11**	2.45±0.14[e]	96.71±2.35[d]	2958.17±171.13[b]	2.12±0.21[e]	4.08±0.48[c]	0.41±0.07[f]	1.99±0.12[d]	1.33±0.32[b]	22.27±6.17[d]
**AMAR20**	2.54±0.11[e]	169.56±10.44[e]	4075.77±151.18[c]	2.34±0.32[e]	6.66±0.85[d]	0.77±0.11[e]	2.03±0.14[d]	1.13±0.51[b]	20.60±4.99[d]
**AMAR25**	2.94±0.15[f]	113.78±8.17[f]	5225.35±190.06[d]	11.66±1.376[f]	10.95±1.40[e]	1.64±0.40[g]	7.00±0.17[e]	1.17±0.51[b]	34.3±5.80[d]

a–g: different superscript letters in the same column indicate significantly different values for a given parameter ($p < 0.05$ by post hoc Tukey's HSD test); same superscript letters in the same column indicate not significantly different values for a given parameter ($p > 0.05$ by post hoc Tukey's HSD test).

Table S4. Volatile compounds (mg/L) of Amarena wines during bottle aging. Data were expressed as mean ± standard deviation of n=15 samples coming from 5 bottles, where every sample was analyzed three times.

Analyte	AMAR1	AMAR2	AMAR3	AMAR4	AMAR6	AMAR11	AMAR20	AMAR25
**Acetaldehyde**	26.19±1.31^{a}	22.49±0.96^{b}	36.74±1.72^{c}	41.91±1.83^{d}	50.01±1.75^{e}	68.89±1.18^{f}	79.37±1.08^{g}	87.17±1.49^{h}
**Ethyl acetate**	40.56±0.96^{a}	41.83±0.84^{a}	38.27±1.17^{b}	55.01±1.02^{c}	93.60±1.27^{d}	86.25±1.06^{e}	96.56±0.84^{f}	102.87±1.87^{g}
**Methanol**	50.69±1.16^{a}	53.49±1.45^{b}	53.94±1.43^{b}	60.66±1.88^{c}	53.55±1.13^{b}	56.02±1.77^{c}	59.59±1.52^{c}	60.51±1.56^{c}
Higher alcohols								
**Propan-1-ol**	31.73±1.33^{a}	51.66±1.28^{b}	39.81±2.02^{c}	40.21±1.94^{c}	40.49±1.87^{c}	39.35±2.13^{c}	36.84±2.23^{c}	37.52±1.61^{c}
**Isobutanol**	62.89±1.81^{a}	60.69±1.52^{a}	50.52±1.20^{b}	61.27±1.51^{a}	72.51±1.66^{c}	69.19±1.81^{c}	80.52±1.97^{d}	81.82±2.15^{d}
**Butan-1-ol**	0.72±0.13^{a}	0.68±0.12^{a}	0.69±0.13^{a}	0.86±0.06^{a}	1.04±0.16^{b}	2.16±0.23^{c}	2.00±0.11^{c}	2.07±0.12^{c}
**2-methyl-1-butanol**	29.63±1.76^{a}	29.30±1.65^{a}	23.48±1.66^{b}	27.52±1.60^{a}	38.99±1.16^{c}	40.29±1.39^{c}	50.23±1.73^{d}	49.99±1.20^{d}
**3-methyl-1-butanol**	179.67±4.24^{a}	177.24±4.16^{a}	182.00±4.46^{a}	176.71±3.38^{a}	180.50±4.72^{a}	190.43±3.52^{b}	199.12±5.34^{b,c}	203.35±4.44^{c}
**Total**	304.64±18.31^{a}	319.57±17.98^{a,b}	296.50±9.31^{a}	306.57±9.71^{a}	333.53±11.21^{b,c}	341.42±29.23^{b,c}	368.71±28.21^{c}	374.75±19.29^{c}
Furanic compounds								
**2-furfural**	0.89±0.39^{a}	0.84±0.35^{a}	1.33±0.32^{a}	6.54±1.25^{b}	2.23±0.80^{c}	9.60±0.99^{d}	22.94±2.06^{e}	30.35±2.17^{f}
**5-hydroxymethylfurfural**	6.06±1.08^{a}	6.55±1.28^{a}	6.11±1.40^{a}	28.70±1.60^{b}	7.83±0.90^{a}	29.67±1.33^{b}	75.72±1.60^{c}	95.54±3.10^{d}
**5-methylfurfural**	0.062±0.015^{a}	0.026±0.012^{b}	0.045±0.017^{b}	0.12±0.03^{c}	0.19±0.07^{c}	0.31±0.03^{c,d}	0.33±0.04^{d}	0.27±0.01^{c,d}
**Furfuril alcol**	0.071±0.02^{a}	0.21±0.04^{b}	0.16±0.03^{b}	0.17±0.02^{b}	0.21±0.04^{b,c}	0.26±0.04^{c}	0.28±0.04^{c}	0.28±0.03^{c}
Ethyl esters								
**Ethyl propanoate**	0.13±0.02^{a}	0.22±0.03^{b}	0.17±0.02^{a}	0.21±0.01^{b}	0.17±0.01^{a}	ND	ND	ND
**Ethyl butanoate**	0.062±0.016^{a}	0.097±0.017^{a}	0.10±0.02^{a}	0.10±0.01^{a}	0.080±0.006^{a}	ND	ND	ND
**Ethyl caproate**	0.10±0.03^{a}	0.13±0.02^{a,b}	0.17±0.02^{b}	0.15±0.01^{a,b}	0.19±0.01^{b}	ND	ND	ND
**Ethyl laureate**	0.090±0.020^{a}	0.11±0.01^{a}	0.19±0.01^{b}	0.18±0.01^{b}	0.19±0.01^{b}	ND	ND	ND
**Ethyl myristate**	0.22±0.03^{a}	0.24±0.03^{a}	0.22±0.01^{a}	0.27±0.02^{b}	0.19±0.01^{a}	ND	ND	ND
**Ethyl palmitate**	0.061±0.008^{a}	0.043±0.011^{a,b}	0.079±0.009^{a}	0.11±0.01^{c}	0.10±0.01^{c}	ND	ND	ND
**Ethyl levulinate**	0.031±0.006^{a}	0.029±0.005^{a}	0.033±0.003^{a}	0.048±0.004^{b}	0.045±0.007^{b}	ND	ND	ND
**Diethyl glutarate**	0.040±0.007^{a}	0.033±0.006^{a,b}	0.025±0.004^{b}	0.061±0.009^{c}	0.033±0.006^{b}	ND	ND	ND

a–h: different superscript letters in the same row indicate significantly different values for a given parameter ($p < 0.05$ by post hoc Tukey's HSD test); same superscript letters in the same column indicate not significantly different values for a given parameter ($p > 0.05$ by post hoc Tukey's HSD test).
